# Image-guided percutaneous ablation for the treatment of lung malignancies: current state of the art

**DOI:** 10.1186/s13244-021-00997-5

**Published:** 2021-04-29

**Authors:** Alfredo Páez-Carpio, Fernando M. Gómez, Gemma Isus Olivé, Pilar Paredes, Tarik Baetens, Enrique Carrero, Marcelo Sánchez, Ivan Vollmer

**Affiliations:** 1 Department of Radiology, CDI, Hospital Clínic, University of Barcelona, Barcelona, Spain; 2 Department of Radiology, The Netherlands Cancer Institute, Amsterdam, The Netherlands; 3 Department of Nuclear Medicine, CDI, Hospital Clínic, University of Barcelona, Barcelona, Spain; 4 Department of Anesthesiology, Hospital Clínic, University of Barcelona, Barcelona, Spain

**Keywords:** Non-small cell lung carcinoma, Metastatic lung disease, Percutaneous thermal ablation

## Abstract

Image-guided percutaneous lung ablation has proven to be a valid treatment alternative in patients with early-stage non-small cell lung carcinoma or oligometastatic lung disease. Available ablative modalities include radiofrequency ablation, microwave ablation, and cryoablation. Currently, there are no sufficiently representative studies to determine significant differences between the results of these techniques. However, a common feature among them is their excellent tolerance with very few complications. For optimal treatment, radiologists must carefully select the patients to be treated, perform a refined ablative technique, and have a detailed knowledge of the radiological features following lung ablation. Although no randomized studies comparing image-guided percutaneous lung ablation with surgery or stereotactic radiation therapy are available, the current literature demonstrates equivalent survival rates. This review will discuss image-guided percutaneous lung ablation features, including available modalities, approved indications, possible complications, published results, and future applications.

## Key points

- Image-guided percutaneous lung ablation is a technique equivalent to surgery and stereotactic radiation therapy to treat patients with early-stage non-small cell lung cancer or oligometastatic lung disease.
- Modalities available include radiofrequency ablation, microwave ablation, and cryoablation.
- Careful selection of patients amenable to ablative treatment is essential, with the tumor's size being the most critical variable.
- CT and PET/CT play an essential role in the immediate and long-term follow-up of patients treated with percutaneous ablation.

## Background

Lung cancer has long been the leading cause of cancer incidence, with around 2.1 million new cases each year and cancer-related mortality worldwide, representing close to 1 in 5 (18.4%) cancer-related deaths [1]. Similarly, the lung is the second most common site for metastases from other malignant tumors [2]. Furthermore, the widespread use of chest computed tomography (CT) has dramatically increased the early detection of potentially treatable lung tumors [3]. Many advances have been made in the diagnosis and treatment of malignant lung tumors. Although surgical resection remains the gold-standard treatment of early-stage non-small cell lung cancer (NSCLC), the increasing number of patients with comorbidities or other reasons for inoperability has led to an increase in the use of less invasive therapeutic options [3–6]. Moreover, several studies have demonstrated the efficacy of metastasectomy for the treatment of oligometastatic lung disease (OLD) [7, 8]. Thus, as in the NSLCL, minimally invasive techniques have emerged as an option for inoperable patients [9].

In this regard, image-guided percutaneous lung ablation has significantly improved in recent years. Several advances regarding ablative modalities, procedure performance, patient selection improvements, and a greater understanding of the imaging findings observed after the procedure have been accomplished, especially in patients with NSCLC or OLD [9, 10]. Currently, radiofrequency ablation (RFA), microwave ablation (MWA), and cryoablation (CA) are the only percutaneous ablative modalities with proven efficacy and safety in the treatment of lung malignancies [11]. They all have shown reasonable local disease control with acceptable complication rates while preserving lung function [12–14].

This review will discuss the different ablative modalities available, current indications, and the preprocedural management of image-guided percutaneous lung ablation. It will also address the most relevant aspects of treatment follow-up, imaging findings after the procedure, and the results obtained in the most relevant research studies published to date.

## Ablative modalities available for lung ablation

Currently available modalities for image-guided lung ablation include RFA, MWA, and CA. All three are thermal ablation modalities that destroy tumor cells by directly applying extreme temperatures into the tumor and the safety margin. Although some publications mention the possible use of laser-induced thermotherapy (LITT) and irreversible electroporation (IRE), none of them reach the results achieved by the RFA, MWA, and CA [15, 16].

RFA is an electric current-based technique that heats tissue by agitating the electrons at a frequency of around 400 kHz. The ablative effect is produced by actively heating the ablation device, diffusing the temperature progressively and passively into the target lesion, thus elevating tissue temperature up to 60–100° C [17]. An expandable array with an electrode diameter of at least 10 mm larger than the target tumor has shown to ablate tumors successfully, ensuring a recurrence rate of < 10% in tumors with a maximum diameter of < 10 mm [18]. The lung is a very susceptible organ to be treated by means of RFA since the air in the lung parenchyma acts as an insulator and an area of low electrical conductivity, allowing the ablation of a larger volume of tissue for a given energy than any other tissue [11]. As for its limitations, RFA is generally not recommended for the treatment of central tumors near large vessels and hilar structures. Although RFA is classically related to cardiac pacemakers' interference, this is no longer the case when using modern equipment [19].

MWA creates an electromagnetic field around the ablation device that varies between 915 and 2450 MHz, causing water molecules to rotate and, ultimately, heat by friction over the target lesion [20]. MWA produces a more uniform ablation zone, and temperature peaks occur much faster than RFA [21, 22]. However, the theoretical superiority of MWA has not resulted in results significantly different from those already reported for RFA: tumor diameter (> 3 cm) and proximity to a large vessel remain the main factors associated with a higher incidence of incomplete treatment [23]. Nevertheless, MWA may allow the treatment of larger tumors than RFA since tissue impedance does not limit the action of MWA [24, 25].

CA generates sub-zero temperatures forming an ice ball to cover the tumor and safety margin at − 40° C. During CA, liquefied gas such as nitrogen or argon passed through cryoprobes to create temperatures as low as − 190° C. Cytotoxic cell destruction is achieved at temperatures below − 20° C [26]. After the freezing phase, a thawing phase follows by replacing the liquefied gas with helium or internally heating the needle. The whole freezing–thawing process is repeated until obtaining an effective ablation [9, 27]. Structures containing a collagenous matrix, such as blood vessels and bronchial tubes, remain intact after CA. This feature makes it ideal for treating tumors near the pulmonary hilum or major vessels [28]. One limitation of CA is that available protocols describe the need for up to three freeze–thaw cycles to achieve a correct ablative treatment, making the procedure longer than RFA and MWA. Another limitation is a greater complexity when handling the equipment since it requires experience operating argon gas. Also, satisfactory ablative treatment often requires the placement of two to four probes within the target lesion, which increases the difficulty of the procedure [29]. However, one advantage of using multiple probes is customizing the treated area's morphology during the procedure.

Current scientific evidence indicates similar therapeutic results for all three ablative modalities. Therefore, it is necessary to carefully consider the tumor features and the patient's characteristics when choosing the ablative technique. For example, although RFA is a widely available technique with proven efficacy and safety, MWA may be preferable in larger tumors. Lung MWA can produce ablative areas of about 6 cm, compared to 3 cm for RFA. Although MWA may be more effective on tumors near the pulmonary hilum and major vessels since the heat dissipation effect does not affect its therapeutic effect, the ablation volume is difficult to control, which increases the risk of bronchial fistula if used near the pulmonary hilum.

Among the advantages of cryoablation compared to heat-based techniques, there is evaluating the ablation site during the procedure. This feature allows the optimization of the treatment in real-time. Cryoablation is an effective alternative in tumors near the great vessels, airways, pericardium, and subpleural lesions, as it tends to cause less pain to the patient than RFA and MWA. As for its disadvantages, CA is not recommended in patients with coagulopathies since a higher rate and severity of lung bleeding. Higher hemoptysis rates have also been reported compared to heat-based modalities [30] (Table 1).

**Table 1:** Comparison between ablative techniques in lung ablation

	RFA	MWA	CA
Mechanism	Electric current	Electromagnetic field	Argon gas
Temperature	60–100° C	60-150° C	Sub-zero
Ablation zone size	3 cm	6 cm	Less than heat-based modalities
Applicators	Single probe: straight or expandable	Single or multiple probes, straight or with one to three loops	Single or multiple probes (2–3)
Advantages	Widely available and proven Lung is highly susceptible	Uniform ablation zone Larger ablated area No heat-sink effect	Suitable for lesions near large vessels or perihilar Less painful
Disadvantages	Not recommended near large vessels or pulmonary hilum Interferes with the heart's conduction system	Superiority to RFA has not been proven It may cause a higher complication rate	Increased difficulty May cause more lung bleeding
Ideal patient	Peripheral tumor, < 3 cm No pacemaker	Peripheral or central tumors Lesion can be > 3 cm It can be used with pacemakers	Peripheral or central lesion can be > 3 cm No bleeding risk factors

*RFA* radiofrequency, *MWA* microwave, *CA* cryoablation, *CT* computed tomography

## Planning and procedure

### Patient selection

The decision to treat lung malignancies by means of image-guided percutaneous ablation has to be made preferably in a tertiary center by a multidisciplinary team including thoracic surgeons, pneumologists, medical oncologists, radiation oncologists, anesthesiologists, and radiologists with expertise in lung ablation [31]. Currently, the indication for percutaneous ablative treatment includes patients with NSCLC or oligometastatic lung disease.

Current indications for image-guided percutaneous lung ablation in NSCLC are: (1) patients with stage Ia NSCLC with contraindications for surgery or stereotactic radiation therapy (SRT) [32, 33]; (2) medically inoperable stage Ia NSCLC; (3) unresectable local recurrence of NSCLC; (4) patients with multiple and synchronous NSCLC (proven by biopsy or by a history of lung cancer), suitable for definitive ablative treatment; and (5) in association with tyrosine kinase inhibitors (TKI), aiming to control the residual tumor volume [34, 35] (6) Recurrence after surgery or radiation therapy (Table 2).

**Table 2:** Indications for image-guided percutaneous lung ablation

	Level of evidence
*Main indications*	
1. Inoperable stage IA NSCLC	2
2. Oligometastatic colorectal cancer with up to 3 lung nodules (≤ 2 cm) and contraindication to surgery	2
*Alternative indications*	
1. Multiple and synchronous NSCLC suitable for definitive ablative treatment	3
2. Inoperable NSCLS in other stages	3
3. Oligometastatic lung disease from other tumors and contraindication to surgery	3

*NSCLC* non-small cell lung cancer

Indications for image-guided percutaneous lung ablation in OLD are less established. Although no prospective studies compare the available alternatives, current guidelines established by the consensus of multidisciplinary committees place complete surgical resection as the treatment of choice in these patients [6]. However, in patients considered at high surgical risk, percutaneous lung ablation is a feasible option, offering local efficacy similar to surgery in carefully selected patients. Percutaneous ablation is performed mainly in metastasis from colorectal, lung and renal cancer, melanoma, hepatocellular carcinoma, and sarcoma. Patients amenable to ablative therapy should have a maximum of four lesions per lung, all with a maximum tumor diameter of < 3.5 cm [36–39]. Also, percutaneous lung ablation may be proposed as rescue therapy for local recurrence in previously irradiated lung metastasis [40] (Table 2).

Absolute contraindications to image-guided percutaneous ablation include severe lung emphysema with bullae (due to the risk of untreatable fistula and respiratory failure), life expectancy of < 3 months, patients with Eastern Cooperative Oncology Group (ECOG) > 2, non-correctable hemorrhagic diathesis, and the presence of small cell lung carcinoma [41]. Relative contraindications are tumors located near large vessels or pulmonary hilum (< 1 cm) lung function deterioration [42] (Table 3).

**Table 3:** Contraindications for image-guided percutaneous lung ablation

*Absolute*	
Severe pulmonary emphysema with bullae	
Life expectancy of < 3 months	
ECOG scale > 2	
Small cell lung carcinoma	
Non-correctable hemorrhagic diathesis	
*Relative*	
Impaired lung function	
Tumors located near large vessels or hilum (RFA)	
Cardiac pacemakers (RFA)	
Correctable hemorrhagic diathesis	

*ECOG* Eastern Cooperative Oncology Group, *RFA* radiofrequency ablation

### Preprocedural evaluation

Preprocedural evaluation for lung ablation is similar to any surgical or minimally-invasive procedure, with assessment and management of cardiopulmonary and systemic conditions, as well as control of bleeding risk factors. A joint effort between radiology teams and an anesthesiology team specially dedicated to interventional radiology procedures is necessary for a correct evaluation. Therefore, it is highly desirable to schedule a visit with the patient, the nursing team, and both specialists on the day of the procedure to evaluate the risks and explain the procedure. A fluid and effective communication between the team and the patient is essential during this visit. Also, a recent cross-sectional imaging study is imperative before the procedure (maximum 4 weeks old). It will allow the radiologist to assess the tumor's size and location, the proximity of the lesion to critical structures, and lymph node involvement [31, 43].

According to the Society of Interventional Radiology and the Cardiovascular and Interventional Radiology Society of Europe guidelines, percutaneous lung ablation is considered a high-risk bleeding procedure. Hence the necessity to control INR and platelet count values correctly [42, 44]. Moreover, any anticoagulant and antiplatelet used by the patient must be adequately evaluated and adjusted before the procedure [45] (Table 4).

**Table 4:** Management of bleeding risk factors before image-guided percutaneous lung ablation

**Pre-procedure laboratory testing and management**	
*INR:* routinely recommended. Correct to < 1.5	
*aPTT:* routinely recommended in patients receiving UFH. Stop or reverse UFH for values > 1.5 × control	
*Platelet count:* routinely recommended. < 50,000: transfuse	
*Hematocrit:* routinely recommended. No recommended threshold for transfusion	
**Anticoagulant and antiplatelet management**	
*Clopidogrel:* withhold for five days before procedure. Resume the day after procedure	
*Aspirin:* withhold for 3–5 days before procedure. Resume the day after procedure	
*LMWH:* withhold for 24 h or up to two doses. Resume the 12 h after procedure	
*Warfarin:* Withhold five days until target INR < 1.5; consider bridging for high thrombosis risk cases. Resume the day after procedure	

*INR* international normalized ratio, *aPTT* activated partial thromboplastin time, *UFH* unfractionated heparin, *LMWH* low-molecular-weight heparin

Regarding antibiotic prophylaxis, although some authors recommend the use of a single dose of antibiotic prophylaxis before a lung ablation, there is no consensus on this implementation. Some risk factors, such as the presence of a single lung, previously irradiated lung parenchyma, primary tumors, or previously compromised parenchyma, may lead to the use of antibiotic prophylaxis. Protocols include amoxicillin-clavulanate or ofloxacin continued for 3–7 days after ablation [46].

### Anesthesia management

In addition to local anesthesia through the probe path, both conscious sedation, recently named procedural sedation and analgesia (PSA) [47], or general anesthesia are valid options in image-guided percutaneous lung ablation [48]. Therefore, it is essential that an anesthesiologist, preferably one with experience in interventional radiology procedures, be always present during the procedure. The anesthesiologist must be in charge of monitoring the patient, diagnosing and treating possible early complications, and ensuring the patient's analgesia, comfort and immobility.

The indication of which type of anesthesia to use depends on the tumor's location, the procedure's technical difficulty, the patient's cardiopulmonary and systemic conditions. Experience of anesthesiology and radiology teams with the chosen anesthetic technique should also be considered. Although general anesthesia guarantees the patient's immobility during the procedure, resulting in less periprocedural pain and lower procedure interruption rates than PSA, the use of general anesthesia may result in a significant increase of anesthetic-induced immunosuppression [49]. General anesthesia also increases the procedure's total cost and duration and pneumothorax risk when positive pressure is used [50, 51]. PSA's main risks are respiratory depression and patients’ movement during the radiological intervention [47]. Nevertheless, comparative studies between general anesthesia and PSA have not resulted in differences in technical success, feasibility, or complications [31, 52].

### CT protocol

For evident reasons (lung air prevents other imaging techniques), CT is the only available imaging modality to place the ablation probe through the lung into the lesion. CT has three different modalities in this regard: conventional computed tomography-guided technique (CCT), CT-fluoroscopy-guided technique (CTF), and cone-beam CT-guided technique (CBCT). Regardless of modality, any CT-guided procedure has a standard workflow: a preprocedural, an intraprocedural and a postprocedural phase.

**Fig. 1:**
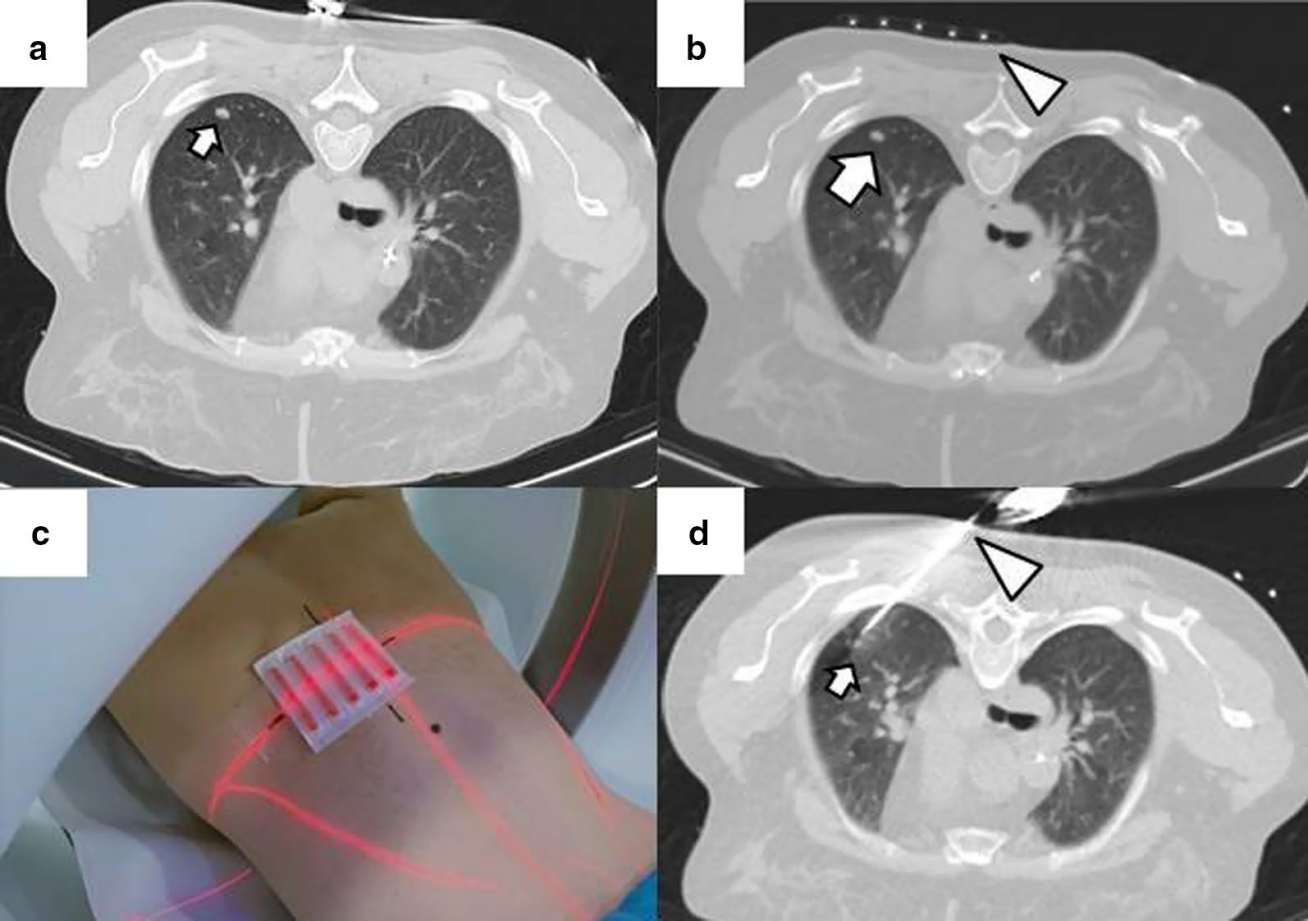
Percutaneous access marking for CTF-guided lung ablation. **a** Preprocedural CT of a patient scheduled for treatment by image-guided percutaneous lung ablation of a single lung colon cancer metastasis located in the left upper lobe (arrow). Note that the CT scan was performed in the position chosen to perform the procedure (prone). **b** Marking of the lesion with a radiopaque grid (triangle). The scanner parameters were modified to decrease the exposure to ionizing radiation of the operator and the patient. Hence the decreased image quality. **c** Example of the marking procedure. The vertical axis is marked with the radio-opaque marker, while the horizontal axis is provided by the CT laser marking (acknowledgment to our resident, Dr. Tomás Fernández, for volunteering for the figure). **d** CTF-guided percutaneous access to the lesion. Note that the access coincides with the point marked in (**b**) (triangle)

Before the procedure, clinical history should be carefully evaluated, in particular previous imaging tests. After acquiring a preprocedural baseline imaging test, the percutaneous access must be marked using a radiopaque grid locator in CCT or CTF or a specific guidance software in the case of CBCT (Fig. 1). Before starting the procedure, the CT acquisition parameters should be adjusted to allow minimum irradiation for the interventional team and the patient while maintaining sufficient quality to perform the procedure without difficulties (Table 5) [53–55].

**Table 5:** Scan parameters recommended for CT-guided lung ablation

kV (range)	80–120 kV
mAs (range)	20–50 mAs
Rotation time	0.5 s
Collimation	1 × 10 mm
CTDI_vol_ unit	CTF: mGy/s CCT: mGy CBCT: mGy and mGy/s
Tube current modulation	Not necessary
Image reconstruction	Eight frames per second
Longitudinal scan length	Smallest possible
Slice thickness	4 mm
Typical accumulated CTDI_vol_	75–100 mGy
Typical accumulated DLP	1000–1200 mGy x cm

CT, computed tomography; CTF; CCT; CBCT; kV, kilovolt; mAs, milliamps; CTDI_vol_, volume CT dose index; DLP; dose-length product

For CTF or CCT, the patient is placed in prone, lateral, or supine decubitus on the CT table, depending on the lesion's location and the optimal access. CCT performs intermittent spiral CT scans that are usually limited to the biopsy. While intermittent spiral CT is being performed, the operator leaves the CT room and is not exposed to radiation [56]. This technique, while preventing radiation exposure to the radiologist, is time-consuming. Also, small subpleural lesions (< 1 cm) are challenging to approach, given their movement with respiration and the impossibility of correcting the probe trajectory in real-time.

Compared to CCT, the procedure time is shorter using CTF. The radiologist directly handles the probe and does not leave the CT room. By manipulating the needle in real-time, subpleural and small lesions are easily approached using this technique. Although CTF may be associated with increased radiation exposure to the operator, if standard radiological prevention measures are used, CTF radiation exposure to the operator does not significantly differ from CCT [57, 58].

In recent years CBCT has gained significance as an alternative to CCT and CBF in image-guided lung procedures. The CBCT system offers advanced needle planning under real-time needle guidance, using a combination of 3D images and fluoroscopy, allowing visualization of the needle's expected trajectory from the skin to the target lesion. Also, the increased workspace provided by the C-arm cone-beam system facilitates needle placement and may speed up the procedure compared to CBF and CCT [59]. Furthermore, CBCT has been shown to have a similar radiological exposure risk to CBF and may be a faster technique than CTF [60, 61].

Regarding the patient's radiation dose, there are several strategies to reduce it to a minimum without compromising the image-guided procedure's quality. Multiple factors contribute to the radiation dose during a CT-guided interventional procedure (Table 5) [55]. One thing to keep in mind is that the preprocedural scan does not have to have the same radiological quality as a diagnostic scan. We can modify the CT parameters to acquire the image with the minimum possible radiation while still being sufficient to perform the procedure. In this regard, several studies have published results of decreased radiation doses to the patient and the operator by modifying CT parameters such as kV, mAs, longitudinal scan length, and slice thickness in CT-guided lung biopsies [53, 62, 63]. Another strategy that has been shown to dramatically decrease patient radiation dose levels during CTF without significantly compromising the results and duration of the procedure is the use of intermittent multislice fluoroscopy rather than continuous single-slice fluoroscopy [56]. Finally, it is advisable to perform a postprocedural acquisition to evaluate the ablation result and rule out immediate complications. The CT parameters should be adequate to make a correct radiological diagnosis without considerably increasing the patient radiation dose [55]".

In addition to conventional radiation protection measures, CTF has different modalities to minimize radiation exposure. Depending on the scanner manufacturer, different softwares are available to reduce radiation exposure. For example, a software widely used in daily clinical practice allows dose reduction and low-dose protocols, such as turning off X-ray emission where it is not needed and reducing the dose to sensitive organs. Another application allows a significant reduction of radiation exposure to the radiologist's hands while performing the procedure. Furthermore, another specific software allows the intervention to be performed with 1 or 3 combined and simultaneous slices, allowing better navigation and reducing the procedure duration and radiation exposure. Finally, other software allows 3D navigation in CT-guided procedures through multiplanar reconstructions via 2D acquisitions [64].

### Procedure

**Fig. 2:**
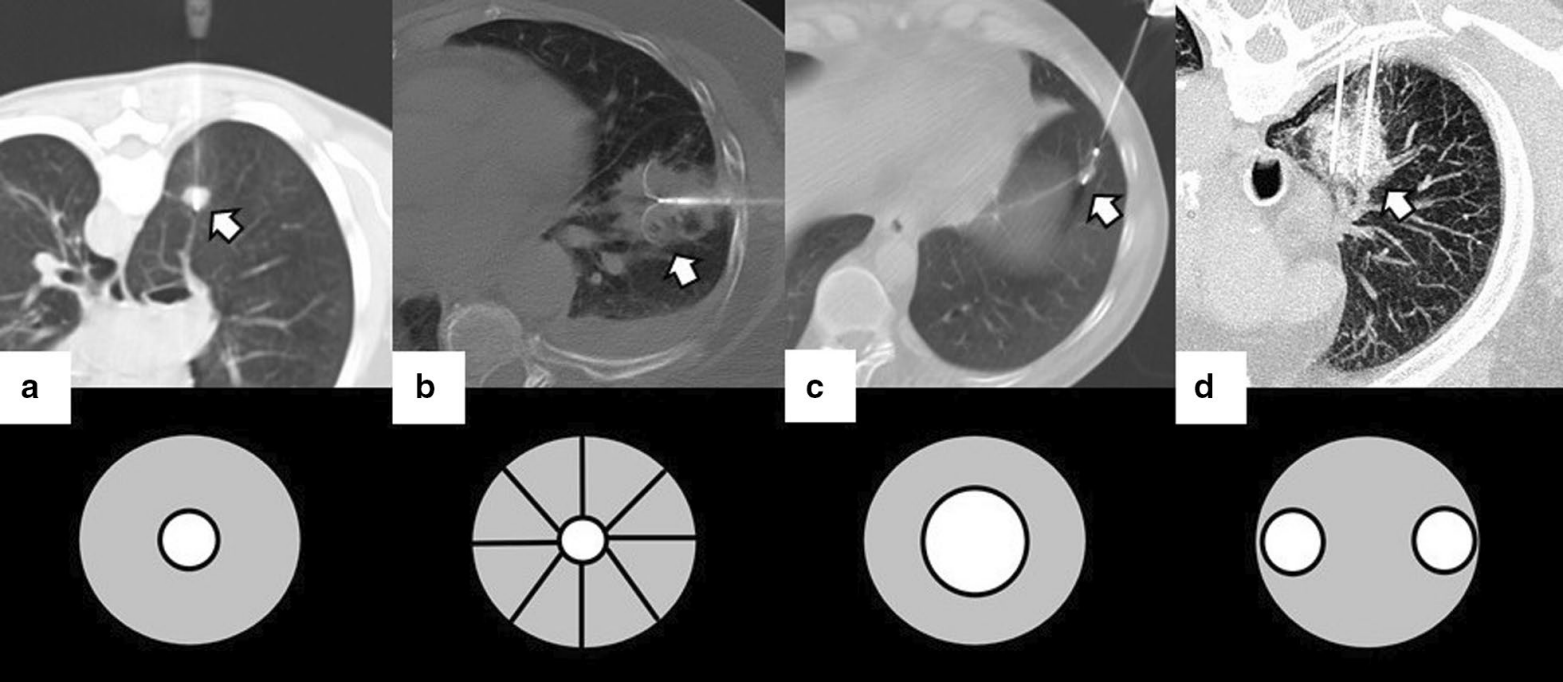
Correct positioning of ablation probes in the target lesion, with schematic illustrations of each ablative alternative. **a** CT-guided percutaneous RFA treatment with a straight needle of a metastatic colon cancer lesion in the lower right lobe. Note the probe's correct positioning in the center of the tumor (arrow). **b** CT-guided percutaneous RFA treatment with an expandable needle of a colon cancer metastasis. The probe’s electrodes include the entire lesion (triangles). **c** CT-guided percutaneous MWA treatment with a single straight needle of a renal cancer metastatic lesion in the lower left lobe. As in (**a**), the probe passes through the center of the tumor (arrow). **d** CT-guided percutaneous CA of a stage I NSCLC in the lower right lobe with two straight ablation probes. If two or more ablation probes are used, they should be placed at the tumor's edges

Depending on the number of probes, the needle should pass through the tumor (single probe) or its edges (multiple probes). However, when expandable probes are used on small tumors, perforating the tumor is not required, as long as one of the deployed arrays includes the tumor (Fig. 2). The electrode must also be at least 10 mm larger than the tumor's maximum diameter [18].

**Fig. 3:**
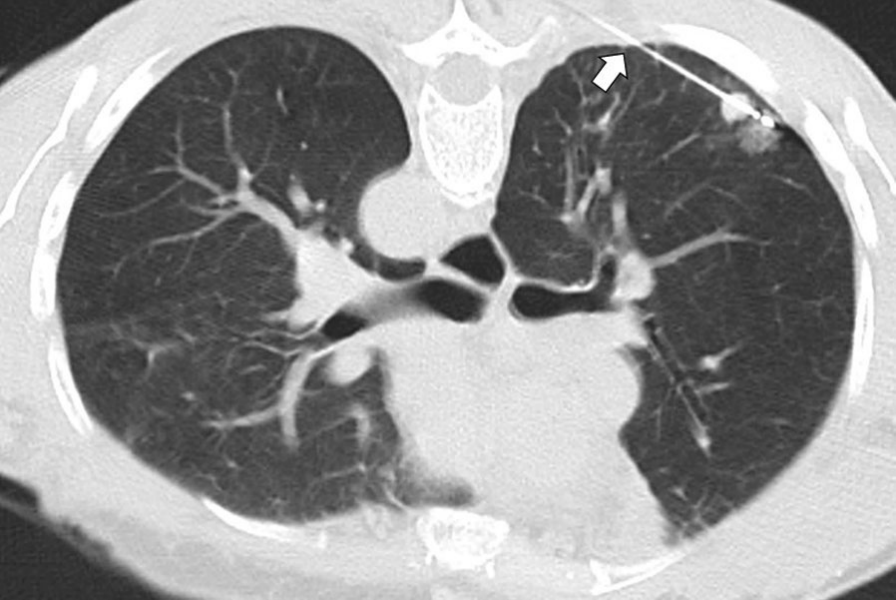
Correct positioning of the ablation probe while treating a subpleural tumor. RFA of a subpleural nodular lesion in the lower right lobe, performed with a straight needle. Note that the needle makes a tangential route to the lesion (white arrow), evading the seemingly easier direct approach (triangle). This approach is necessary to allow a better anchorage in the lung parenchyma, thus avoiding the needle's non-voluntary displacement

**Fig. 4:**
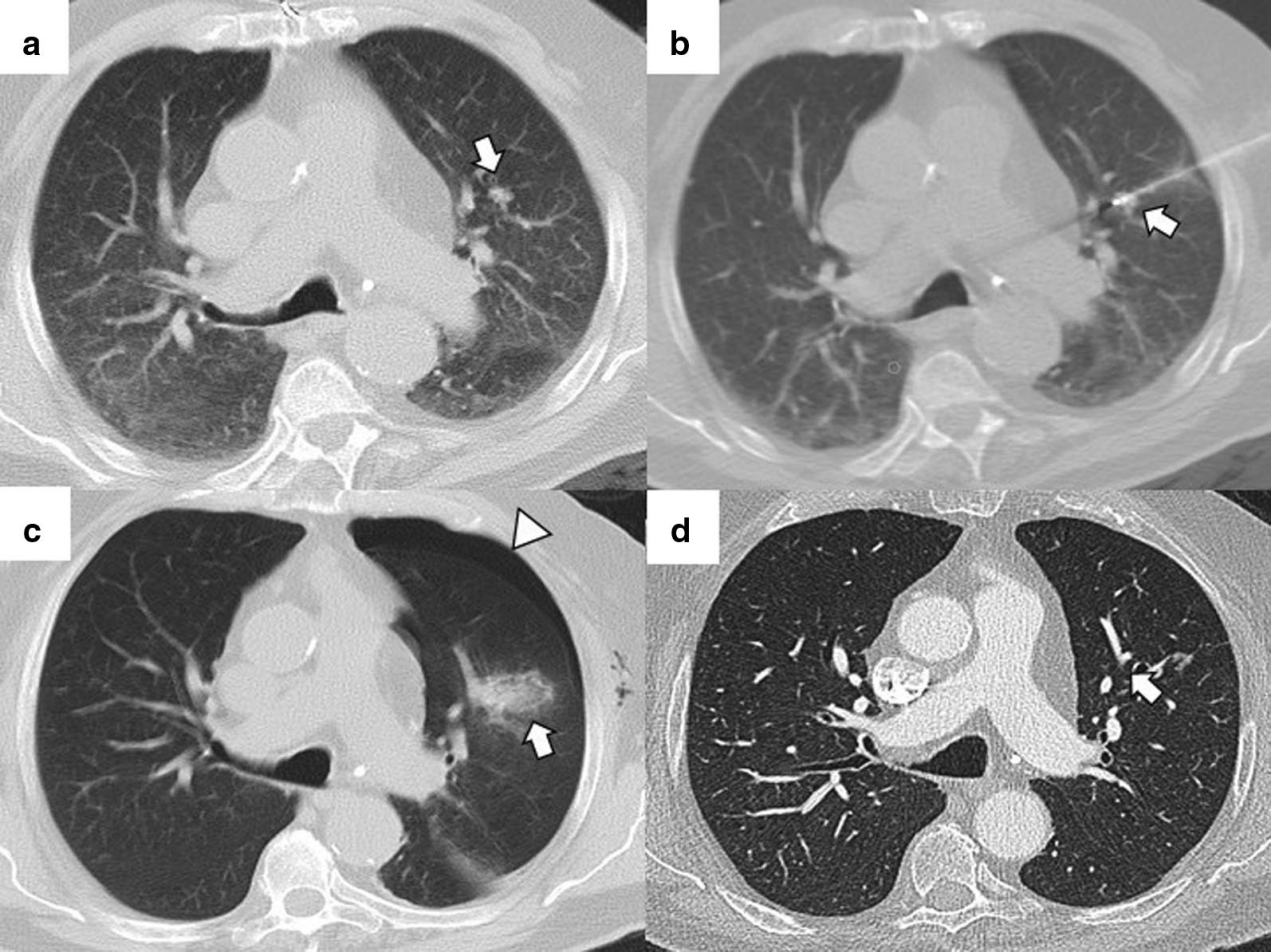
Lung parenchyma retraction while performing an image-guided percutaneous lung ablation. **a**, **b** RFA treatment with a straight needle of a metastatic lesion of a colon adenocarcinoma located in the left upper lobe, very close to the pulmonary hilum (white arrow). **c** After treatment, the treated area presents an image with a consolidative center and a ground-glass peripheral halo, suggesting a complete treatment (white arrow). A discrete retraction of the pulmonary parenchyma is evident, which, added to the presence of a mild pneumothorax (white triangle), mobilizes the hilar structures. **d** Control of the treated lesion after 12 months of RFA, no signs of recurrence are evident

While placing the probe in the target lesion, the tumor’s location and its situation concerning the pleural fissures and bronchovascular structures should be considered. Subpleural lung tumors may be challenging to treat by direct puncture perpendicular to the pleura, as the probe may not be optimally anchored in the lung, thus, moving while breathing. Therefore, it is best to direct the probe tangentially to the pleura to properly anchor it in the lung parenchyma (Fig. 3). However, when using cryoablation during the first freezing cycle, it is possible to attach the probe to the lesion in order to place the second needle, a technique known as stickfreeze [65]. For central tumor ablation, the needle position should be parallel to the adjacent bronchovascular structures. Using CA this risk does not exist, which allows the tip to be directed towards the pulmonary hilum, as this is the area that offers maximum probe control. It is also important to anticipate a decrease in lung volume during ablation, which will cause the ablation probe to end up closer than expected to vital structures [48] (Fig. 4).

In tumors located near sensitive structures (e.g., mediastinum, pleura, great vessels), it is possible to use adjunctive thermoprotection techniques during percutaneous lung ablation. Available techniques include manual traction using the ablation probe, iatrogenic pneumothorax, and hydrodissection. As previously stated, manual traction is possible using expandable electrodes or cryoprobes, as both can archor the tumor during the procedure. The formation of an iatrogenic pneumothorax makes it possible to separate the tumor from the pleura and mediastinum during ablation. Finally, using hydrodissection, a variable amount of solution can be injected into the mediastinum using a small-caliber needle to avoid injuring it while performing the ablation [66].

In the event of treating lesions in both lungs, it is possible to treat them in a single session, provided that the patient meets specific inclusion criteria and that a radiological control is performed between each lung treatment. Given the risk of developing a bilateral pneumothorax. The passage of an electrode through a major pulmonary fissure should also be avoided. Also, the nodules should not be in contact with the pleura in both lungs [67].

A CT scan must be performed immediately after the procedure to rule out severe complications. Depending on the patient's condition, monitoring in a hospitalization room can vary between 24 and 48 h. An additional CT scan is optional during this period. The radiologist must be personally involved in the clinical follow-up of the patient.

## Complications

**Fig. 5:**
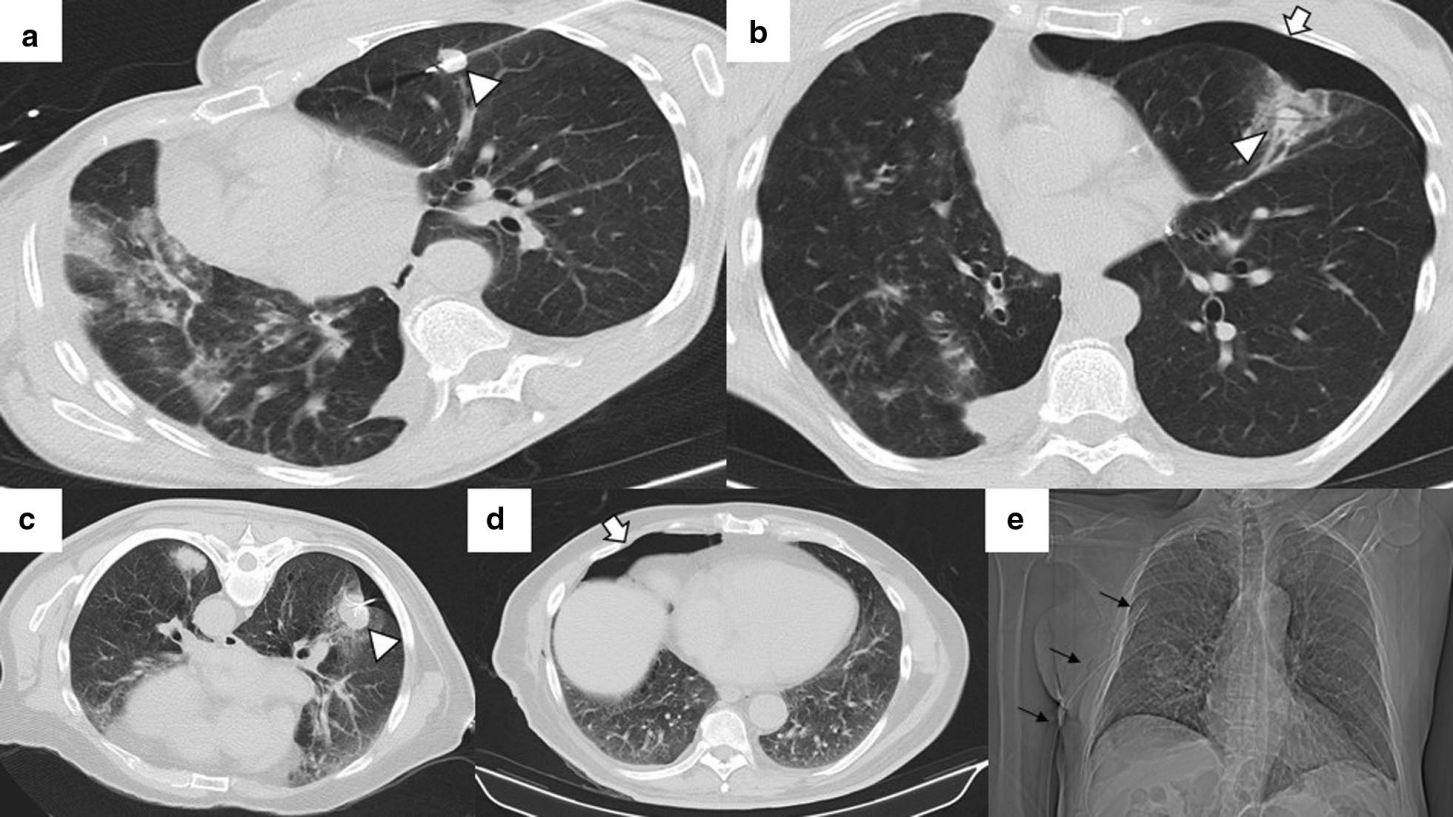
Mild pneumothorax after image-guided percutaneous lung ablation. **a**, **b** CT-guided MWA in a single lung metastasis from colon cancer in the upper left lobe. Note the correct probe position in the center of the lesion (triangle). In the CT performed immediately after the procedure, the patient presented a mild pneumothorax (arrow). The pneumothorax was resolved spontaneously without the need for a chest tube. Note the peripheral ground-glass halo with a central consolidation (triangle in **b**), which indicates a correct treatment. **c**–**e** CT-guided RFA lung ablation in a lung metastasis from a basal cell skin carcinoma. The expandable probe correctly englobes the lesion (triangle in **c**). In this patient, a pneumothorax was also observed immediately after the ablation. (arrow in **d**). Although pneumothorax is similar to in A-B, the patient's clinical situation required a chest tube (black arrows in **e**)

Regardless of the ablation mechanism, pneumothorax is the most frequent immediate complication following image-guided percutaneous lung ablation. Rates vary between 30 and 60%, and most are asymptomatic, successfully managed mostly with clinical monitoring and sequential chest x-rays (Fig. 5) [68, 69]. However, about 30% may have symptoms or an increase in size. Most improve promptly after the placement of a chest tube [10].

A rare complication is a late pneumothorax, defined as a pneumothorax occurring as early as 4 h after the procedure, almost always due to a bronchopulmonary fistula. This complication is also usually managed by placing a chest tube [69, 70]. MWA may be associated with an increased risk of developing bronchopulmonary fistulas, possibly due to higher temperatures during the procedure and a larger ablation area [71].

The incidence of small asymptomatic pleural effusions after ablative treatment is not uncommon and is most likely a reactive phenomenon [31]. The vast majority are asymptomatic and resolve spontaneously. However, even the slightest pleural effusion may warrant thoracentesis or even a chest tube placement in patients with low respiratory reserve. Thoracentesis or chest tube placement is required between 1 and 7% of the patients treated [69].

**Fig. 6:**
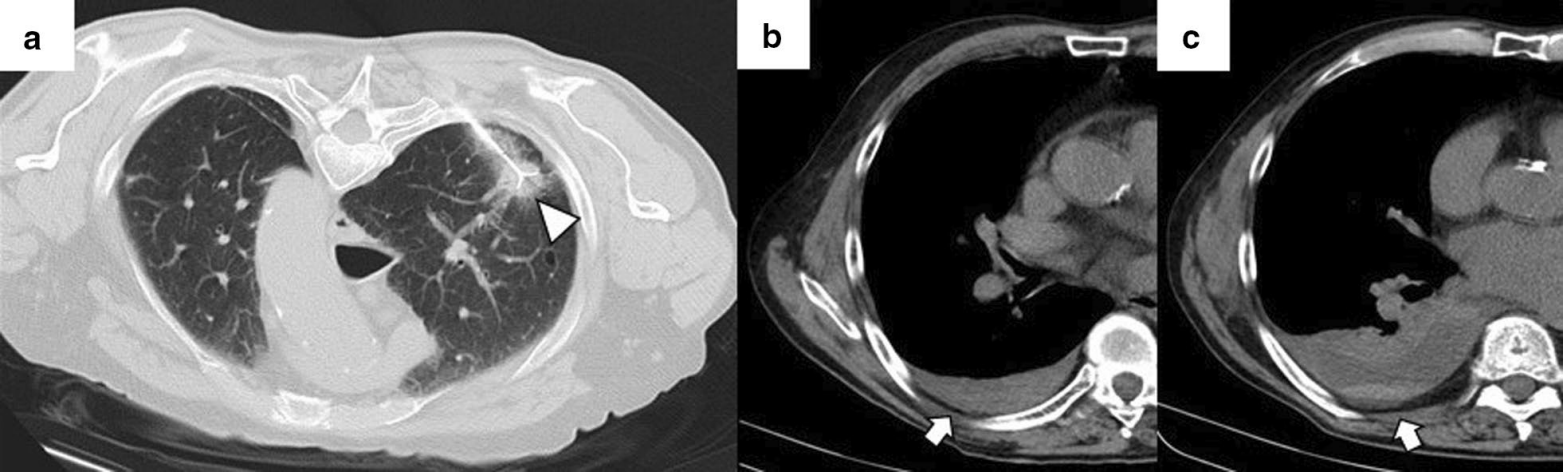
Mild hemothorax after image-guided percutaneous lung ablation. **a** CT-guided percutaneous RFA of a stage 1 NSCLC located in the lower left lobe. Note the expanding tube encompassing the entire lesion. **b** CT scan immediately after the procedure where we observe a mild hyperdense pleural effusion, consistent with a mild hemothorax. **c** CT scan performed 24 h after the procedure shows a slight increase in the hyperdense pleural effusion and a discrete hyperdense level within the pleural effusion, confirming the hemothorax (arrow). However, after 48 h of clinical and radiological stability, the patient was discharged

**Fig. 7:**
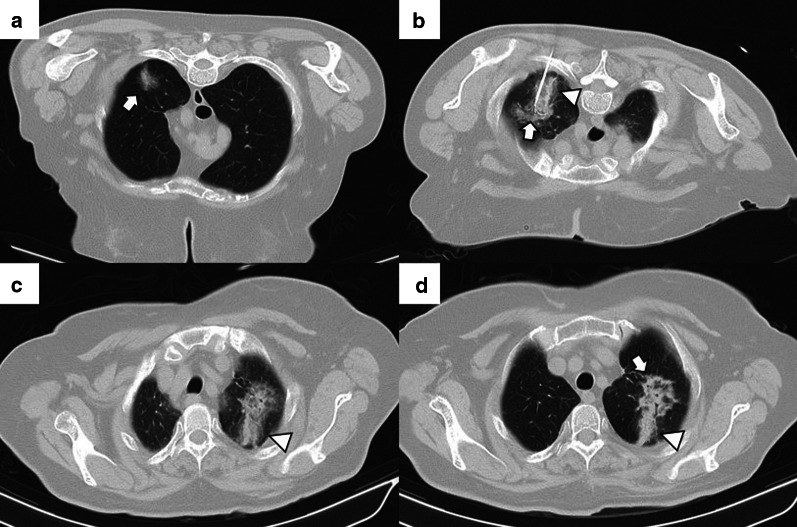
Mild lung hemorrhage in the path of the ablation probe. **a**, **b** CT-guided percutaneous RFA with an expandable probe of a stage 1 NSCLC (arrow in **a**). The expandable probe completely covers the tumor in **b** (arrow), and the formation of a lung hemorrhage is visible in the probe’s path (triangle). **c** In the CT scan performed immediately after the procedure, we can observe a mild lung hemorrhage related to the probe’s path (triangle). **d** The lung hemorrhage remains stable in the CT 24 h after the procedure, and the patient remains asymptomatic. Note the presence of a ground-glass halo englobing a consolidative center, a suggestive sign of a correct ablative treatment (arrow in **d**)

The development of a hemothorax is a rare complication after percutaneous lung ablation, but it can be fatal if left untreated. We should suspect it in the event of a rapidly progressive pleural effusion, especially if there are signs of hypovolemia. It requires a radiological examination or thoracentesis to confirm the diagnosis (Fig. 6) [69]. Although most cases require only a chest tube, endovascular treatment may be necessary if active arterial bleeding is reported [31]. Parenchymal hemorrhage is another common complication after percutaneous lung ablation. The vast majority are self-limiting and are mostly related to the probe's path through the lung (Fig. 7). Some evidence indicates a higher rate of severe lung hemorrhage after CA [72].

Much rarer complications include the development of pneumonia or the presence of chronic obstructive disease exacerbation, both reported with the use of RFA [70]. Another sporadic complication is the injury of adjacent critical structures. Such structures include the nerve, mediastinum and diaphragm. A safe distance should always be taken between these structures and the probe, considering the lung parenchyma's possible retraction during the procedure [10]. Finally, an infrequent complication related only to CA is the development of shock secondary to an uncontrolled release of cytokines, also called cryoshock. This is usually related to the volume of ablation [72]. Finally, complications, depending on severity, may prolong hospital stay and costs.

## Postablation imaging follow-up

Patients treated by percutaneous ablation must follow a strict and standardized scheme to detect and treat an incomplete ablative treatment since, unlike surgical resection, the treated zone remains in the lung parenchyma. CT is the modality of choice for radiological follow-up, although positron emission tomography (PET/CT) is becoming increasingly common. A recommended scheme for follow-up after treatment is as follows: the patient is monitored by a contrast-enhanced CT at 1, 3, 6, 9, 12, 18, 24 months, and annually afterward [42]. Depending on availability, it is advisable to perform a PET/CT scan at 3 and 12 months after treatment and whenever there is a suspicion of recurrence on CT [73, 74] (Table 6).

**Table 6:** Follow-up Scheme after Image-Guided Percutaneous Lung Ablation

Pretreatment	1 month	3 months	6 months	9 months	12 months	18 months	24 months	Yearly
CE-CT	CE-CT	CE-CT	CE-CT	CE-CT	CE-CT	CE-CT	CE-CT	CE-CT
PET/CT		PET/CT			PET/CT			PET/CT*

*CE-CT*, contrast-enhanced computed tomography; *PET/CT*, positron emission tomography/computed tomography

*In case of suspected tumor recurrence

We will discuss the morphological and metabolic changes visualized in the treated area during the radiological control, subdivided into four distinct phases: after-treatment-phase (< 24 h), early-phase (< 24 h to 1 month), intermediate-phase (1 to 3 months), and late-phase (> 3 months) (Table 7).

**Table 7:** Radiological Follow-up after Image-Guided Percutaneous Lung Ablation

	After-treatment-phase	Early-Phase	Intermediate-phase	Late-phase
Successful treatment	Consolidative center surrounded by a peripheral ground-glass area > 5 mm	The peripheral ground-glass opacity evolves into a thin consolidative area The resulting consolidation is typically more extensive than the original tumor	Progressive decrease in the size of the treated area Persistence of a benign periablational enhancement, yet less than the original tumor	Three to six months after ablation: stability in size. After six months: progressive decrease in size Increased enhancement compared to previous phases may exist
Unsuccessful treatment	Irregular peripheral nodular enhancement within the consolidative center	Excessive growth of the treated area Irregular and peripheral enhancement or within central consolidation	PET/TC: solitary focal or peripheral FDG uptake or combined with a focal uptake at the consolidative center	Enlargement of the treated area after the first three months that persists beyond six months Any FDG uptake suggests recurrence

After-Treatment-Phase: < 24 h; Early-Phase: 24 h to 1 month; Intermediate-Phase: 1 to 3 months; Late-Phase: > 3 months; PET/TC: Positron Emission Tomography/Computed Tomography; FDG: fluorodeoxyglucose

### After-treatment phase (< 24 h)

**Fig. 8:**
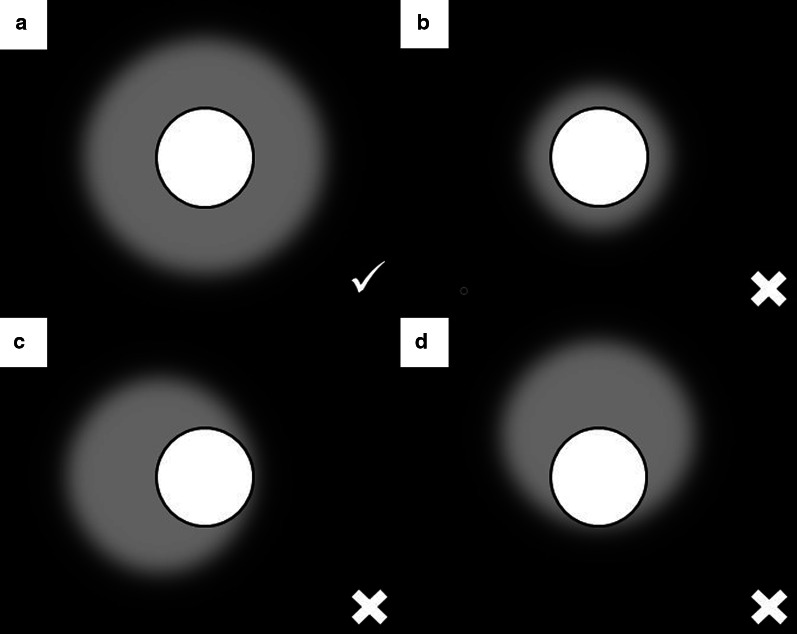
Schematic illustration demonstrating the ground-glass halo's correct disposition surrounding the treated area. **a** Proper disposition of the ground-glass halo after ablation lung treatment. The halo must completely surround the tumor and be greater than 5 mm, preferably between 8 and 10 mm. **b**–**d** Different forms of incomplete treatment after percutaneous pulmonary ablation as demonstrated by the disposition of the ground-glass halo

**Fig. 9:**
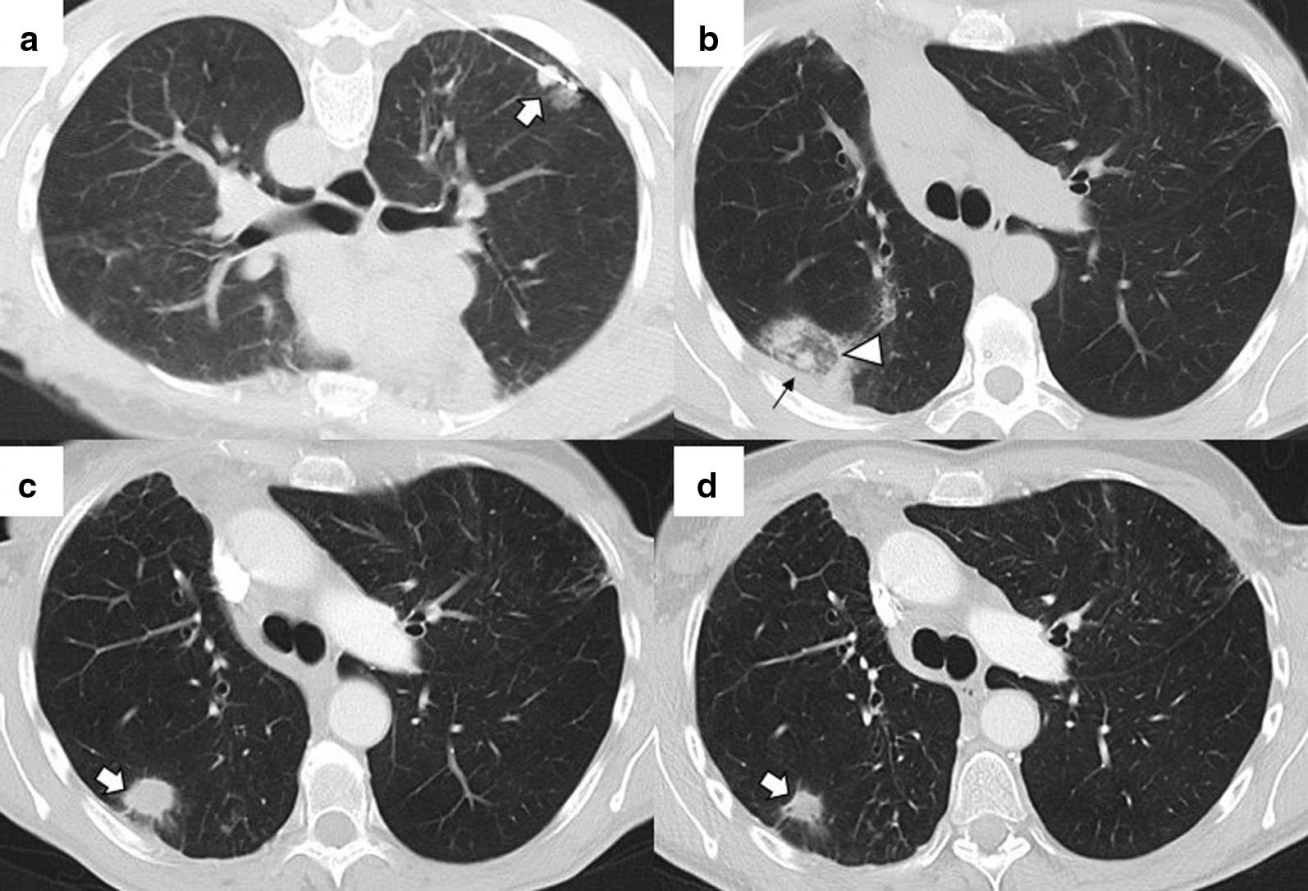
Radiological findings indicative of successful treatment immediately following image-guided percutaneous lung ablation. **a** CT-guided MWA with a single straight probe of a stage 1 NSCLC located in the lower right lobe. **b** We observed a consolidative center (black arrow) surrounded by a peripheral ground-glass halo (triangle) in the area treated in the CT scan performed immediately after completion of the procedure. The combination of these findings is indicative of successful treatment. **c** Follow-up CT one month after treatment. Note the increase in the size of the treated area compared to A. However, there are no irregular nodular areas nor other signs of tumor persistence. **d** CT 6 months after treatment The treated area has decreased in size compared to C, suggesting a successful treatment

**Fig. 10:**
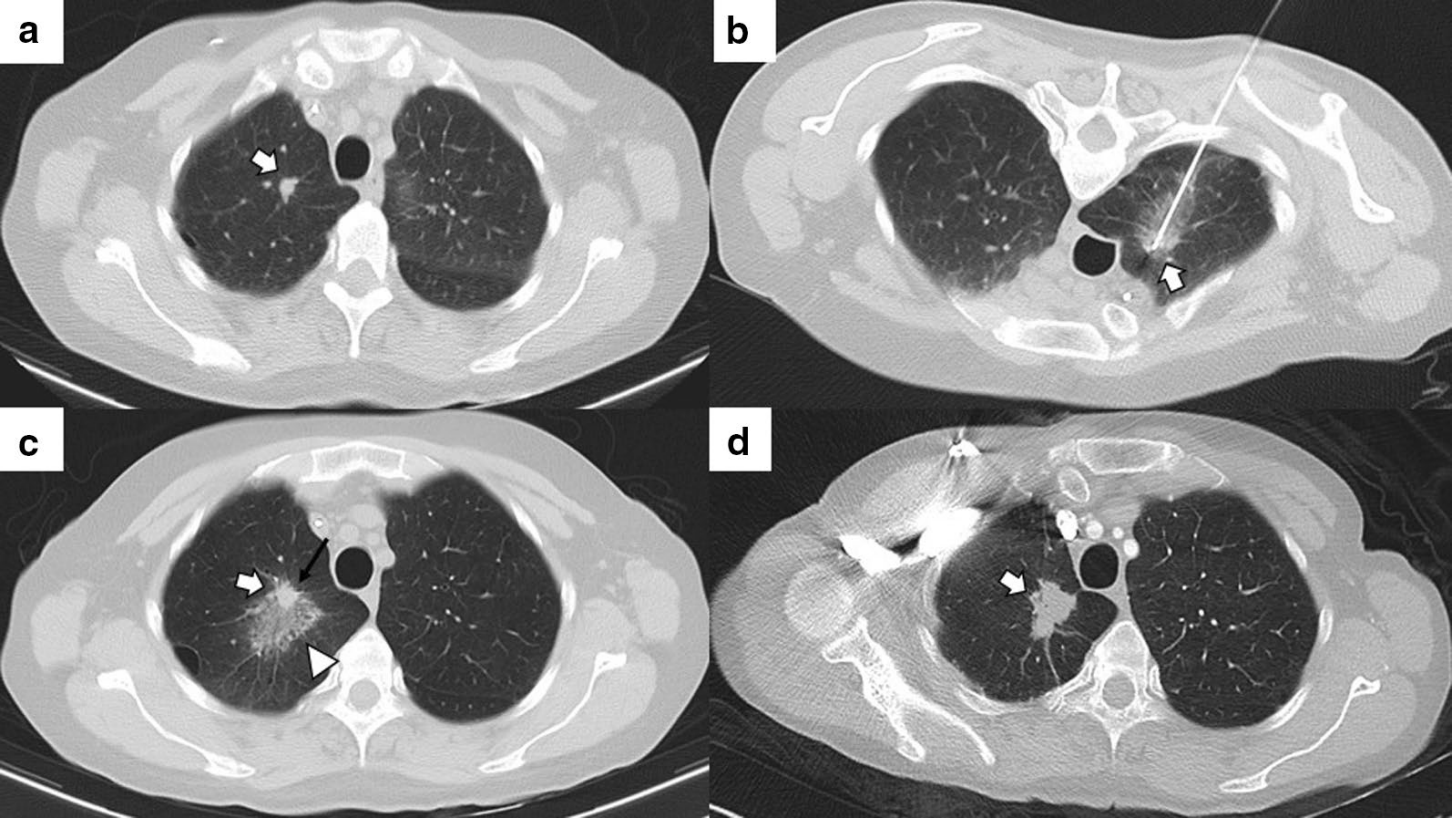
Tumor persistence after treatment with image-guided percutaneous lung ablation. **a** Non-enhanced CT showing a single metastatic tumor in the upper right lobe in a patient with rectal carcinoma. **b** CT-guided RFA treatment using a straight probe, passing through the center of the tumor. **c** A non-enhanced CT was performed 24 h after treatment, showing the treated lesion (thick white arrow) surrounded by a ground-glass halo (triangle). However, the treated tumor's frontmost portion does not present a ground-glass halo (thin black arrow), which may correspond to incomplete treatment. **d** Contrast-enhanced CT scan performed three months after treatment shows a considerable increase in the treated lesion, which now presents nodular and irregular borders, consistent with tumor persistence

**Fig. 11:**
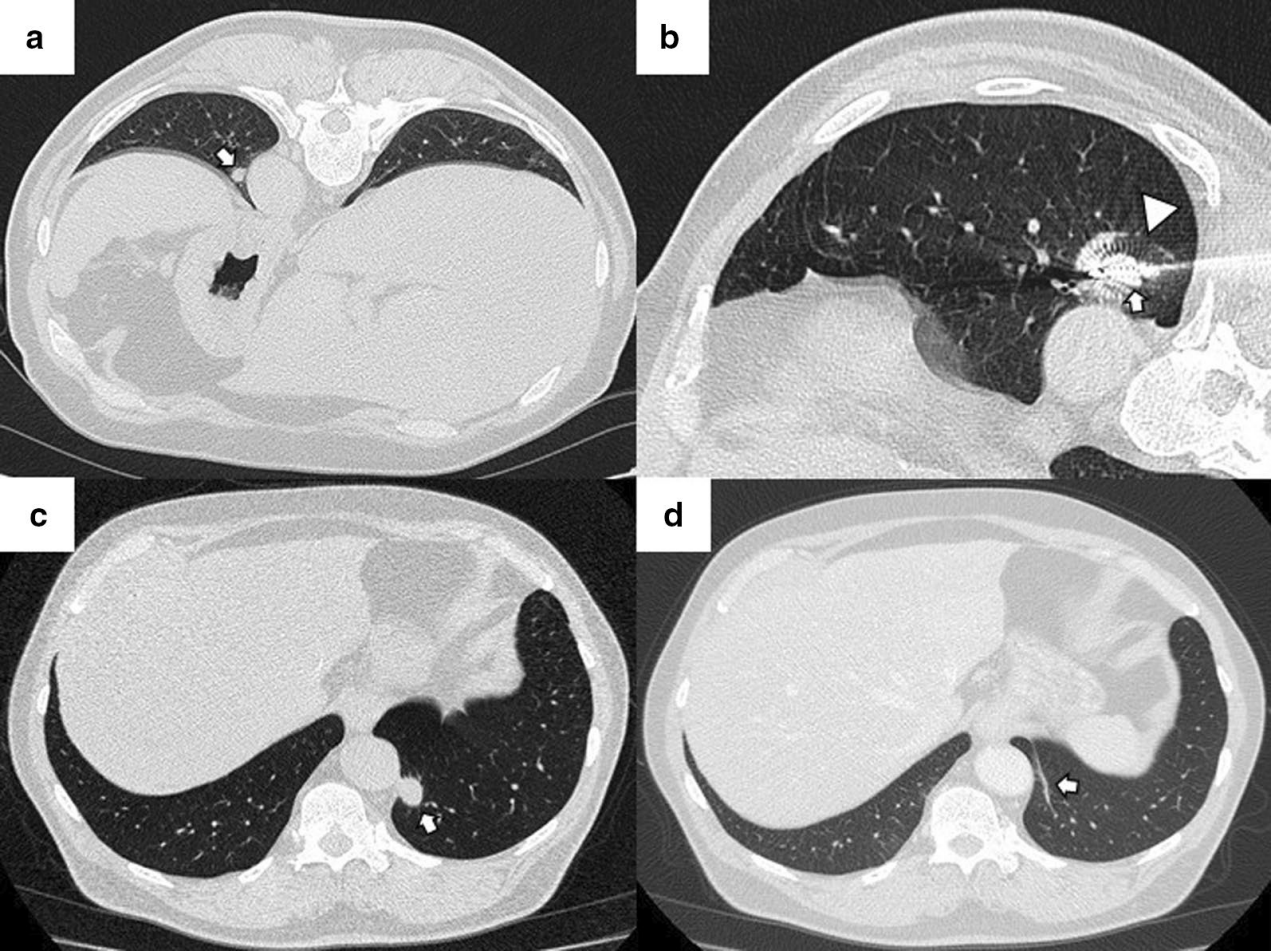
"Iceball" formation while performing an image-guided percutaneous lung CA. **a** CT of a patient with oligometastatic disease due to a small cell renal carcinoma with a lesion near the descending thoracic aorta (arrow). **b** CT-guided CA of this lesion, given the lesion's small size, it was possible to use a single straight probe placed in the center of the lesion (arrow). Note the hypodense halo surrounding the lesion, consistent with the “ice ball” formed during treatment (triangle). **c** Non-contrast CT scan one month after treatment. The treated area shows a larger size than the original lesion (arrow). However, it is a rounded area of similar size to the image immediately after treatment (not available). **d** Non-contrast CT scan six months after treatment. A lung scar is now observed in the treated area, consistent with effective treatment (arrow)

**Fig. 12:**
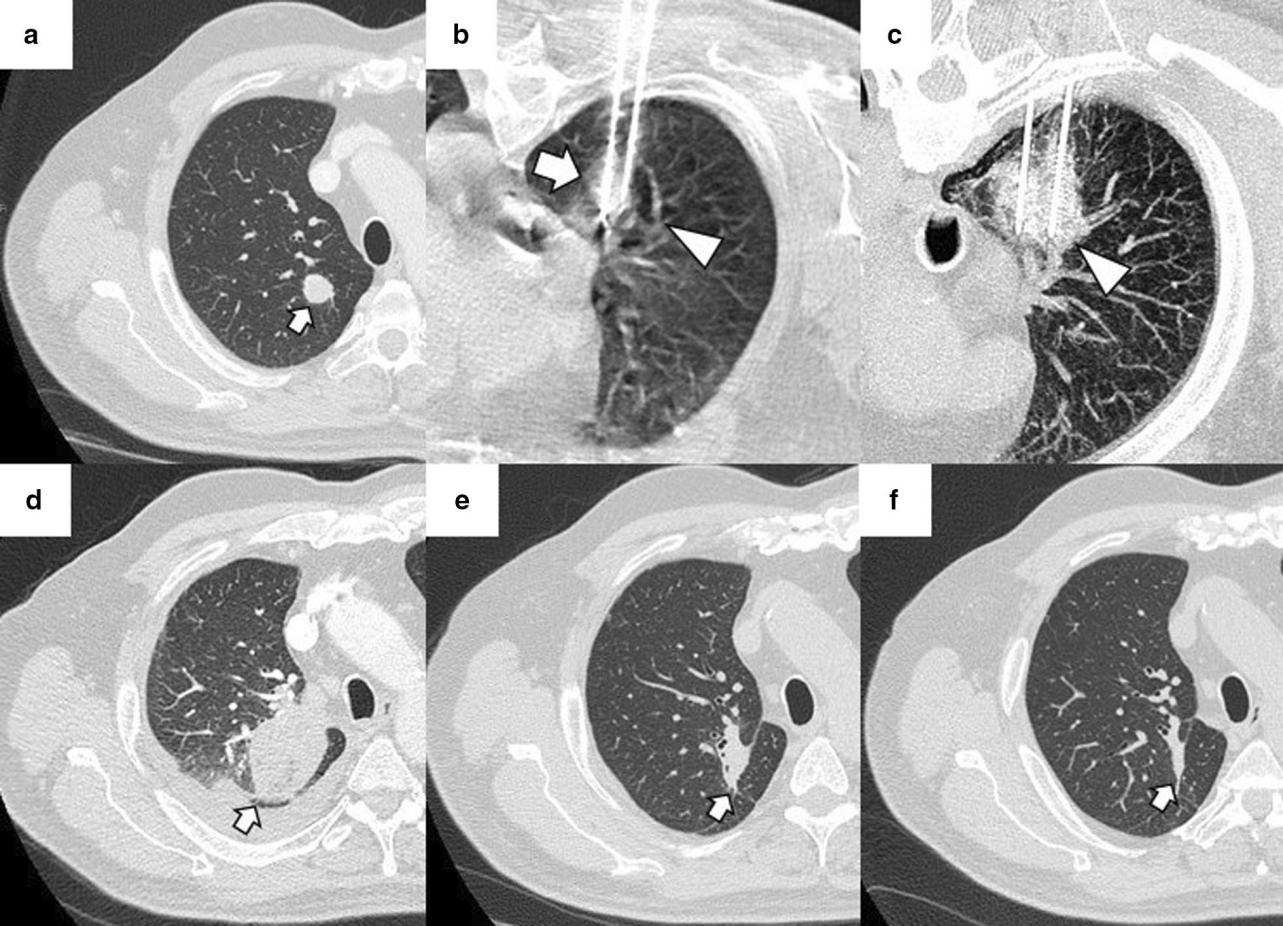
"Iceball" formation and further evolution to an area of lung hemorrhage in an image-guided percutaneous pulmonary cryoablation. **a** CT scan performed before ablative treatment, demonstrating a single metastatic lesion from a colon carcinoma in the upper right lobe (arrow). **b**, **c** CT-guided CA performed with two straight probes. Note the position of the probes, parallel and at the edges of the lesion. In (**b**) the ice ball (triangle) is observed surrounding the tumor (arrow). This ice ball was subsequently replaced by a ground-glass halo in (**c**) (triangle). **d** Contrast-enhanced CT scan performed one month after treatment, with a treated area more extensive than the original lesion (arrow). **e**, **f** The treated area gradually decreases in size at three (**e**) and six months (**f**) after treatment, consistent with successful treatment (arrows)

The most common finding in RFA and MWA immediately after the procedure is a pattern consisting of a ground-glass peripheral halo surrounding a central consolidation. The presence of a ground-glass halo greater than 5 mm around the ablated zone suggests treatment success (Figs. 8 and 9) [75]. In contrast, the persistence of an irregular and enhancing nodular zone within the central consolidation is consistent with incomplete treatment. Other signs of incomplete ablation are the total or partial absence of the ground-glass peripheral area and the extension of such halo by less than 5 mm (Fig. 10) [76]. CA offers the advantage of differentiated the ablated area and the surrounding hemorrhage throughout the procedure, the former represented as a low attenuation zone ("ice ball") and the latter as a higher attenuation zone (Fig. 11). Towards the end of the procedure, the melted ice induces necrosis, hemorrhage, and edema, which manifest as a peripheral ground-glass halo surrounding a central consolidation, very similar to that observed in heat-based ablations (Fig. 12) [30].

### Early-phase (24 h to 1 month)

**Fig. 13:**
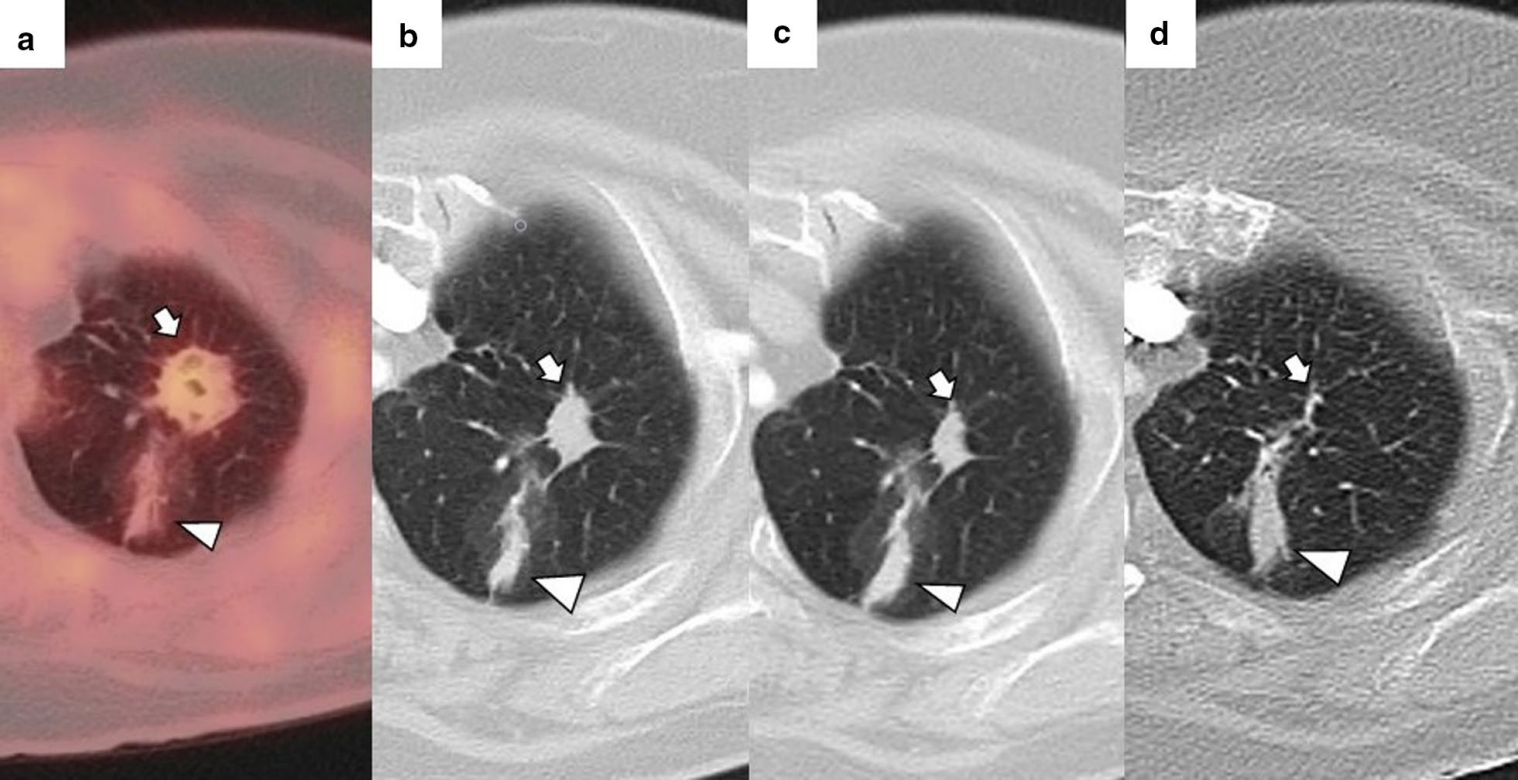
FDG uptake in a PET/CT one month after image-guided percutaneous lung ablation. **a** PET-CT acquired after one month of percutaneous lung MWA, showing a growth of the ablative area compared to the pre-treatment CT (Fig. 7-A). It also presented a subtle uptake of FDG within the treated area (arrow). Given the proximity of the treatment, this is probably due to an inflammatory reaction. We also observe a residual scar due to a past probe-path-related lung hemorrhage reported (triangle). **b–d** We observed a progressive reduction in the size of the ablative area after 3 (**b**), 6 (**c**), and 12 months (**d**) of treatment, finally observing a residual scar (arrows)

In all ablative modalities, as the weeks progress, the ground-glass opacity surrounding the ablation site gradually resolves and is replaced by a thin residual consolidation zone that separates the central consolidation from the adjacent lung. The resulting consolidation is typically more extensive than the original tumor (Fig. 9c) [77]. At this stage, CT is the only technique available for radiological control. PET-CT has limited value due to the inability to differentiate fluorodeoxyglucose (FDG) uptake due to inflammation from that caused by a residual tumor (Fig. 13) [78]. Using CA, diffuse internal enhancement may occasionally occur, which resolves within a month [79].

### Intermediate-phase (1 to 3 months)

**Fig. 14:**
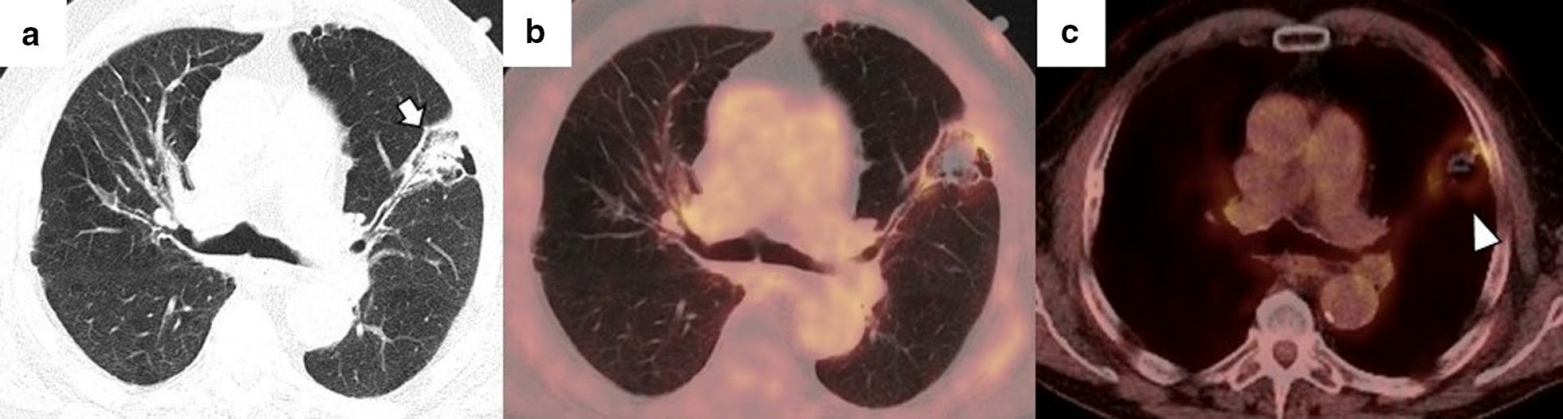
Peripheral FDG capture pattern on a PET/CT performed two months after image-guided percutaneous lung ablation. **a** non-contrast CT showing the treated area: a central consolidation surrounded by a peripheral ground-glass opacity with a tendency to consolidate (arrow). **b**, **c** PET/CT demonstrating peripheral FDG uptake of the treated area, a pattern suggestive of response to treatment

**Fig. 15:**
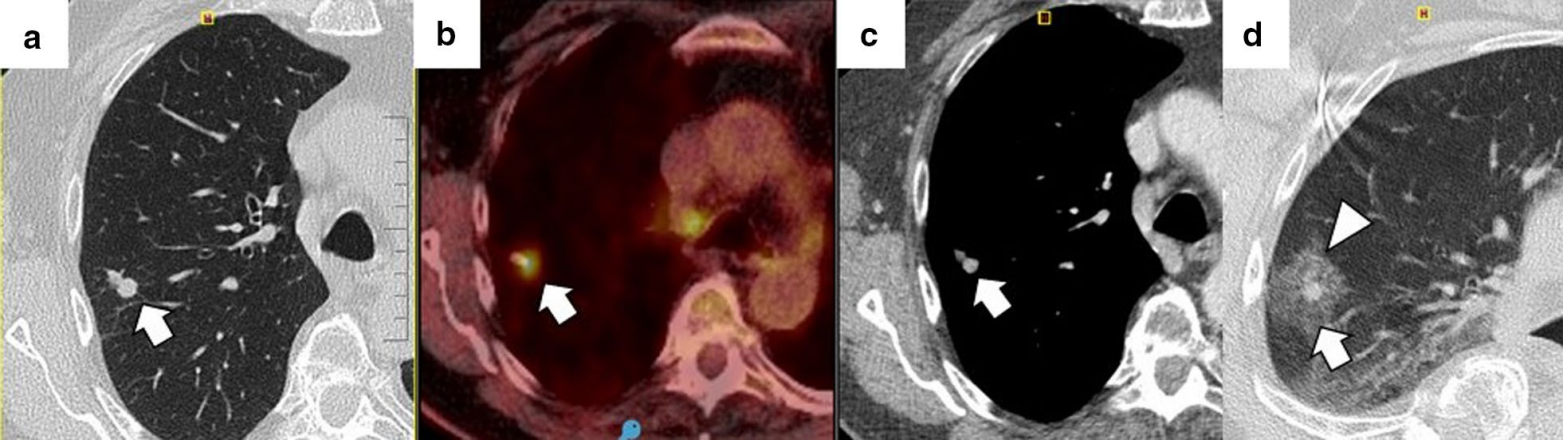
Diagnosis and retreatment of a tumor recurrence previously treated by image-guided percutaneous pulmonary RFA. **a–c** Appearance of a solid nodular lesion adjacent to an ablative area in the radiologic control performed six months after a percutaneous lung RFA to treat lung metastasis from a rectal carcinoma (arrow). This lesion exhibits avid FDG (**b**) and iodine contrast (**c**) uptake (arrows), and it is consistent with a tumor recurrence at the ablation site. **d** The lesion is re-treated using percutaneous lung MWA, with a central consolidative area (arrow) surrounded by a ground-glass halo of more than 5 mm (triangle). These findings are consistent with comprehensive treatment

Given the regression of edema, inflammation, and hemorrhage, the ablated area should have decreased in size compared to the size observed immediately after the procedure. The enhancement at this phase should be less than the original tumor, with a benign periablational enhancement persisting for up to 6 months [80]. In some cases, especially in large lesions, central cavitation secondary to the drainage of necrotic tissue by the adjacent bronchi can be seen throughout this phase [81]. Pleural thickening and a transient size increase in the hilar and mediastinal lymph nodes also occur during this phase [48]. FDG uptake peaks two weeks after ablation and should return to regular activity two months after the procedure. The following FDG uptake patterns suggest treatment response: diffuse, peripheral, heterogeneous, and peripheral plus focal at a different site than the initial lesion (Fig. 14). A solitary focal or a diffuse peripheral uptake combined with a focal uptake at the same site of the original lesion suggests tumor progression/recurrence (Fig. 15) [82, 83].

### Late-phase (> 3 months)

**Fig. 16:**
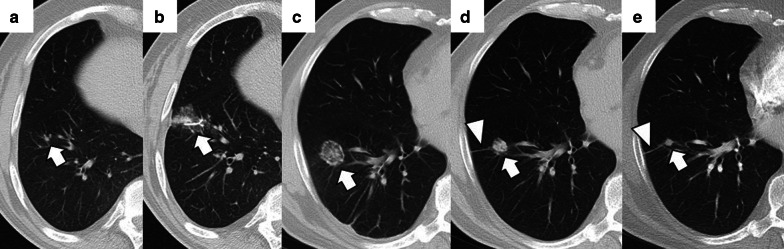
Evolution towards a nodule pattern after successful treatment with image-guided percutaneous lung ablation. Non-enhanced CT showing a small metastatic lung tumor from a rectal carcinoma in the lower right lobe (arrow in **a**). The CT scan performed 24 h after treatment with lung RFA shows a central consolidating area surrounded by a ground-glass opacity at the site of ablation (arrow in **b**). Although the ablative area is larger than the original tumor in the radiologic control performed one month after treatment, it has decreased in size compared to the post-ablative image (arrow in **c**). After six months of treatment, only a residual consolidation of rounded morphology remains in the treated area (arrow in **d**), with an adjacent lung scar (triangle in **c**, **d**)

**Fig. 17:**
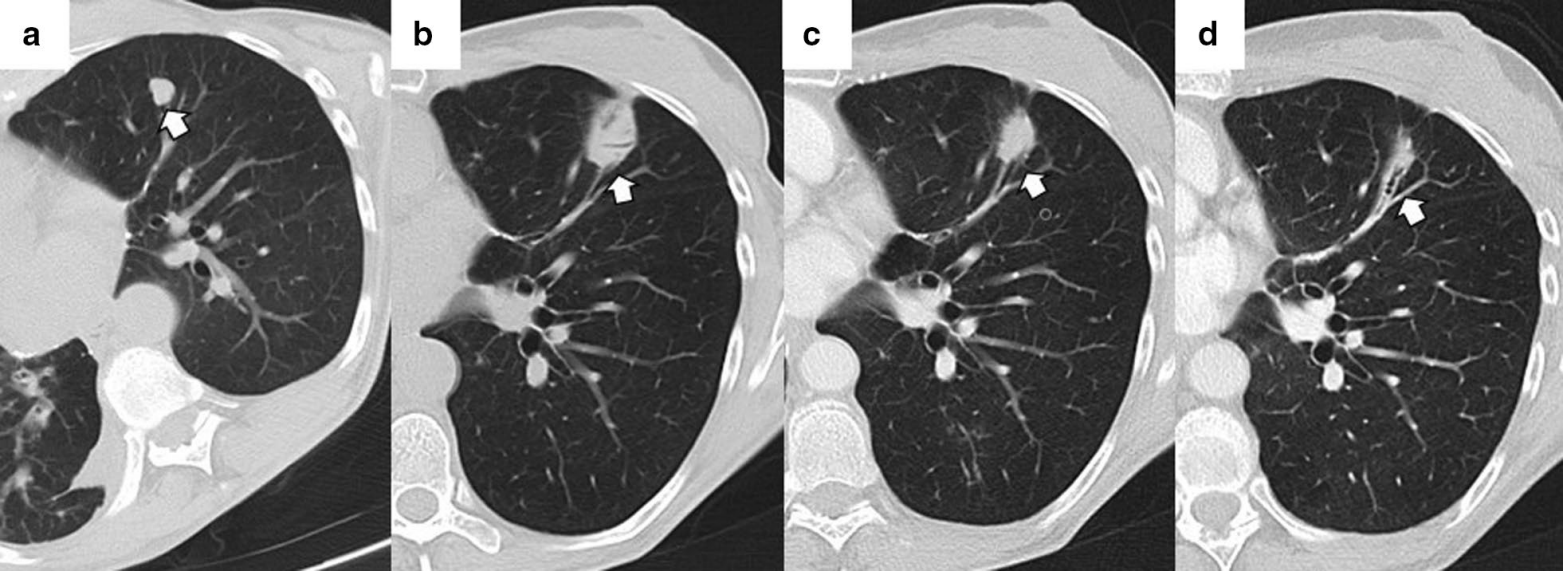
Imaging follow-up with an evolution towards fibrosis after successful treatment with image-guided percutaneous lung ablation. CT showing a metastatic lung lesion of < 2 cm due to a small cell renal carcinoma (arrow in **a**). The treated area has grown in size compared to the original tumor in the radiological control after one month of treatment (arrow in **b**). However, in the radiological follow-up performed following three (**c**) and six months (**d**) after treatment, it successively reduces its size until only an area of fibrosis remains (arrow in **d**)

**Fig. 18:**
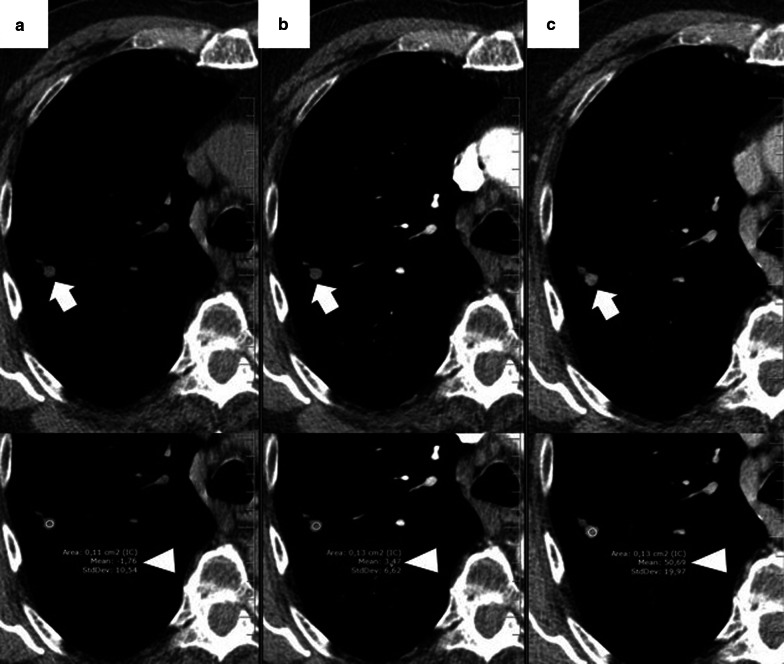
Contrast enhancement in a nodule adjacent to the treated area after image-guided percutaneous lung ablation. **a** Unenhanced CT showing a nodular lesion adjacent to an area treated with a lung ablation (arrow). **b**, **c** The lesion enhances after administering intravenous iodine contrast (arrows), successively increasing HU between the arterial and venous phase (triangles). These findings are compatible with tumor recurrence

During the first 3 to 6 months, the treated area should be stable in size. After this period, the zone will progressively decrease in size, reaching a size smaller than that of the original tumor. During this period, the treated area should present a morphology that varies between ovoid, rounded or linear, and, eventually, a lung scar (Figs. 16 and 17). After treatment, the treated area may evolve into five distinct patterns: fibrosis (most common), nodular pattern, cavitation, atelectasis, and local tumor progression. The presence of none of the first four has been shown to predict the occurrence of tumor progression [75]. However, an increase in the treated area's size after the first three months that persists beyond six months after ablation suggests tumor recurrence [79]. Due to the recovery of microcirculation in the ablation area in the first three months after ablation, there may be an increase compared to the initial or intermediate period, which should gradually decrease in the following three to six months. Nonetheless, at no time should enhancement exceed that of the original tumor (Fig. 18) [48]. After two months, any hypermetabolic activity observed by PET-CT within the ablation site suggests tumor progression/recurrence [83].

## Clinical outcomes

### Stage I NSCLC

Numerous studies have documented the safety and efficacy of RFA in patients with stage I NSCLC. The first published retrospective studies reported overall survival rates (OS) after RFA treatment at 1-, 2-, 3-, 4-, and 5-year of 78%, 57%, 36%, 27%, and 27% and local recurrence rates of 12% at one year, 18% at two years, and 21% at three years, respectively. Furthermore, these early studies already reported that tumors whose diameter was < 3 cm showed the best results, with a higher rate of recurrence in tumors whose maximum diameter was > 2 cm [84, 85]. The first published prospective multicenter trial evaluated 54 patients with stage Ia NSCLC treated with RFA. This study reported OS rates of 86.3% after one year and 69.8% at two years, including OS rates of up to 83% in tumors with a maximum diameter < 2 cm [74]. A recent prospective multicenter phase II trial involving 32 patients with stage Ia NSCLC not suitable for surgery and treated with RFA showed local control rates of 84.38% and 81.25% and OS rates of 91.67% and 58.33% at 1 and 3 years. Furthermore, this study reported no significant changes in lung function after treatment [73]. These studies showed that the maximum tumor diameter is the most critical characteristic for predicting technical and therapeutic success. Tumors with a maximum diameter between 2 and 3 cm seem to be more amenable to successful treatment. Moreover, we can observe a progressive increase in OS rates between early published studies and those more recently conducted. This increase is probably due to a progressive improvement in both the ablation technique and patient selection.

Although not as extensively studied as RFA, MWA is gaining increasing popularity and acceptance in image-guided percutaneous lung ablation. Much like RFA, the first studies published at MWA were noncomparative retrospective studies. Two of the most important reported 1-, 2-, 3-, and 5-year OS rates between 78–89%, 54–63%, 39–43%, and 16% and 1-, 3-, and 5-year local control rates of 96%, 64%, and 48% [86, 87]. Also, the authors of one of such studies reported a mean time to recurrence at 39.7 months. This last parameter showed a strong correlation with the maximum diameter of the treated tumor. Thus, tumors with a maximum diameter > 3 cm showed a median time to recurrence of 17.3 months, while those with a maximum diameter of < 3 cm showed a median time to recurrence of 62.1 months [86, 87]. Another recent retrospective study compared MWA with RFA in 161 patients with lung tumors, of which 41 had a phase 1 NSCLC (18 treated with RFA and 23 treated with MWA). This study reported similar efficacy and safety between the two techniques. However, this study fails to specify the recurrence and survival rates segmented by tumor type [25]. No prospective studies comparing both ablative techniques are currently available.

Unlike MWA and RFA, there are currently only a handful of significant studies evaluating the utility of CA in the treatment of NSCLC. One of the first relevant studies published evaluated retrospectively 45 patients with medically inoperable stage I NSCLC. This study reported a 5-year OS rate of 67.8% with a 5-year progression-free survival rate of 87.9%. Local recurrence rate was 36.2% [88]. Additionally, in a more recent retrospective study, 25 patients with stage I NSCLC treated with CA showed 1 and 3 year OS rates of 100% and 63%. However, local control rates were 71% at one year and 37% at three years, both lower than those reported using MWA or RFA. Like MWA and RFA, the maximum tumor diameter appears to be the most critical predictive parameter. Tumors with a maximum diameter of > 3 cm are associated with a higher risk of progression [89]. Although these studies may show that CA has similar efficacy to RFA in the treatment of stage I NSCLC, these are retrospective studies with very few patients. Prospective and comparative studies are needed to establish the real value of CA early-stage NSCLC.

In the largest retrospective series published to date, OS rates of thermal ablation and SRT were compared in a sample from the National Cancer Database in the United States. All three lung ablation modalities were considered. Thermal ablation proved to be non-inferior compared to SRT. OS rates at 1-, 2-, 3-, and 5-year were 85.4%, 65.2%, 47.8% and 24.6% for thermal ablation and 86.3%, 64.5%, 45.9% and 26.1% for SRT [32].

### Alternative indications in NSCLC

Alternative indications for image-guided percutaneous lung ablation include treatment of local recurrence after treatment with radiotherapy and in combination with chemotherapy at inoperable NSCLC in advanced stages. A retrospective study in which 12 patients with local recurrence of NSCLC after treatment with radiotherapy and chemotherapy received rescue therapy using percutaneous thermal ablation (RFA/MWA) reported a median time to recurrence of 14 months and an OS of 35 months. In such patients, we must consider the increased risk of developing bleeding complications due to radiation-associated vasculopathy when treating these patients [40]. Furthermore, a recent prospective noncomparative study that evaluated the utility of percutaneous ablation in patients with stage IIIB or IV NSCLC after treatment with first-line chemotherapy reported a mean local control time of 10.6 months and an OS of 17.7 months [90].

The advent of immunotherapy drugs for lung cancer treatment is one of the most novel developments in recent years in the treatment of NSCLC [27]. In this regard, recently published studies have investigated the combination of percutaneous ablation with immunomodulatory therapy. A prospective randomized study compared the effect of combining CA with Gefitinib, an orally active epidermal growth factor receptor-TKI (EGFR-TKI), with Gefitinib alone in patients with advanced NSCLC. This study reported superior partial regression and disease stabilization and decreased disease progression rates in the CA + Gefitinib group compared to those that received only Gefitinib [35]. Moreover, a prospective controlled study demonstrated the safety and an increase in survival of CA combined with allogenic natural killer (NK) cell immunotherapy for the treatment of advanced NSCLC compared to the control group (CA alone), with no significant increase in adverse effects [91].

### Oligometastatic lung disease

As in the NSCLC, RFA has been the most extensively tested and documented technique in OLD percutaneous ablative treatment. One of the most extensive retrospective studies published to date evaluated 566 patients with 1037 metastases from various primary tumors. This study reported a median OS of 62 months, a 5-year OS of 52%, and local tumor progression rates of 5.9%, 8.5%, 10.2%, and 11.0% 1-, 2-, 3-, and 4-year [68]. Also, as with NSCLC, tumors with a maximum diameter < 3 cm showed improved local tumor response and higher OS. Primary tumor type, disease-free interval, and the presence of more than three lung metastases were also significant variables [68]. The first prospective study to evaluate RFA treatment in patients with OLD (RAPTURE study) reported 1- and 2-year OS rates between 89–92% and 64–66% [92]. Furthermore, a recently published retrospective study evaluated 188 patients with lung metastases from colorectal carcinoma treated with RFA. This study reported a median progression-free survival of 6.8 months and an OS of y 52.7 months. Likewise, among the variables that influenced the OS, the most remarkable were: the presence of extrapulmonary metastases and maximum tumor size of > 15 mm [39]. In the most important (prospective and multicenter) and recently published study on this subject, Hasegawa et al. reported a series of 70 patients with lung metastases from colorectal cancer of < 3 cm treated with RFA an OS rate at 3 years of 84%, with hardly any complications (1% of severe complications). Factors associated with a worse OS include a rectal rather than a colon origin and the absence of chemotherapy [93].

Unlike NSCLC, several published studies have evaluated the value of MWA in OLD. One of the first prospective studies published evaluated 80 patients with unresectable lung metastases treated with MWA. At 12 and 24 months, the OS rates were 91.3% and 75%. Furthermore, as in RFA, size was the main predictor of therapeutic success, with incomplete ablation more likely in lesions > 3 cm. There was also a better technical success in peripheral lesions than those close to the pulmonary hilum [94]. Another retrospective study compared MWA, RFA, and LITT results in 109 patients with lung metastases from colorectal carcinoma. Local tumor control rates were 68.0% for LITT, 69.2% for RFA, and 88.3% for MWA, with statistically significant differences between MWA and the other two techniques. The median time of local tumor progression published for each technique was 10.4 months for LITT, 7.2 months for RFA, and 7.5 months for MWA, as well as OS of 22.1 months for LITT, 24.2 months for RFA, and 32.8 months for MWA [95]. A much recent published meta-analysis compared the results obtained between RFA and MWA in the ablative treatment of pulmonary metastases, with a total inclusion of 3,432 patients. Patients treated with RFA presented an OS and a survival rate at 1,- 2,- 3, and 5 years higher than the MWA branch, reporting similar local recurrence rates between the two techniques. However, given the studies' heterogeneity, the high probability of publication bias, especially in the RFA branch, and a lower number of patients in the MWA group, one technique's superiority over the other could not be definitively concluded [16]. In conclusion, MWA has proven to be a technique at least as effective and safe as RFA in OLD's ablative treatment. However, prospective randomized studies comparing both techniques are still needed.

Two single-arm prospective multicenter studies have demonstrated the efficacy and safety of CA in treating pulmonary metastases. In 2015, the ECLIPSE study included 40 patients with 60 lung metastases treated with CA, with a follow-up of at least 12 months. This study applied strict inclusion criteria. Local tumor control rates were 96.6% and 94.2% at 6 and 12 months, respectively. The one-year overall survival rate was 97.5% [96]. The SOLSTICE study, a multicenter, phase II, prospective, single-arm study involving 128 patients with 224 lung metastases treated with CA, recently published its results. The patients' follow-up in this study was from 12 to 24 months, and the inclusion criteria were laxer compared to the ECLIPSE study. After the first ablation, the response rate without local recurrence was 85.1% at 12 months and 77.2% at 24 months. After a second CA for local recurrence treatment, the response rate without local recurrence was 91.1% at 12 months and 84.4% at 24 months. The mean OS rate at 12 and 24 months was 97.6% and 86.6% [97]. The results of both studies demonstrated the efficacy and safety of this technique in the treatment of OLD. Studies comparing CA with the other available ablative techniques, as well as with metastasectomy, should be conducted.

All available evidence recommends, regardless of the ablative technique selected, the need for patient selection guided by strict pre-established clinical and radiological criteria to avoid unnecessary treatment and obtain local tumor progression and OS rates similar to those offered by surgical metastasectomy. Moreover, to design therapeutic algorithms in OLD treatment, it is necessary to conduct prospective and randomized studies that compare percutaneous ablation in its different modalities with surgical treatment.

## Conclusions

Image-guided percutaneous lung ablation has proven to be a safe and effective treatment modality in patients with early-stage NSCLC or OLD. As there are no specific protocols established in handling these patients, there must be a careful patient selection to avoid unnecessary treatments and undesired results. In this regard, size is the most critical factor in predicting ablative treatment success. It is also essential to know the radiological findings observed in the treated area during treatment and follow-up. When choosing among the available ablative modalities, the available scientific evidence indicates that their efficacy and safety are comparable. Therefore, selection depends on the specific characteristics of the tumor and the patient. Finally, prospective, comparative, and randomized studies between these techniques, SBRT, and surgery are pending to define and improve patient selection.

## Data Availability

Data sharing is not applicable to this article as no datasets were generated or analyzed during the current study.

## Abbreviations

CA: Cryoablation
CBCT: Cone-beam CT-guidance
CCT: Conventional computed tomography-guided technique
CT: Computed tomography
CTF: CT-fluoroscopy guided technique
ECOG: Eastern Cooperative Oncology Group
EGFR: Epidermal growth factor receptor
FDG: Fluorodeoxyglucose
IRE: Irreversible electroporation
LITT: Laser-induced thermotherapy
MWA: Microwave ablation
NK: Natural killer
NSCLC: Non-small cell lung carcinoma
OLD: Oligometastatic lung disease
OS: Overall survival
PET/CT: Positron emission tomography
RFA: Radiofrequency ablation
SRT: Stereotactic radiation therapy
TKI: Tyrosine kinase inhibitors
